# Mannose receptor RpMR1 of Manila clam (*Ruditapes philippinarum*) defense against *Vibrio anguillarum* infection

**DOI:** 10.1007/s44307-025-00075-7

**Published:** 2025-08-04

**Authors:** Zhihui Yin, Hongtao Nie

**Affiliations:** 1 College of Fisheries and Life Science, Dalian Ocean University, Dalian, 116023 China; 2 Engineering Research Center of Shellfish Culture and Breeding in Liaoning Province, Dalian Ocean University, Dalian, 116023 China

**Keywords:** *Ruditapes philippinarum*, Mannose receptor, Gene expression, Immune function

## Abstract

The mannose receptor (*MR*) is a member of the C-type lectin superfamily and a type I transmembrane protein that functions as a pattern recognition receptor (PRR) in immune responses. In this study, we identified 13 *MR* genes (RpMR1-13) in the genome of *Ruditapes philippinarum* and investigated their expression profiles following *Vibrio anguillarum* challenge. Notably, *RpMR1*, *RpMR2*, *RpMR3*, and *RpMR4* exhibited peak expression at 72 h post-infection. We successfully purified the recombinant RpMR1 protein and demonstrated its antibacterial activity against three Gram-negative bacteria (*V. splendidus*, *V. alginolyticus*, and *V. anguillarum*), though it had no effect on Gram-positive bacteria. Furthermore, in vivo injection of RpMR1 significantly reduced mortality in *R. philippinarum* following *V. anguillarum* infection. To explore role of *RpMR1* in immune signaling, we performed RNA interference (dsRNA-RpMR1) and observed successful gene silencing. Subsequent qRT-PCR analysis revealed that *RpMR1* knockdown significantly suppressed *TLR4* expression (*P*< 0.05) under *V. anguillarum* stress, confirming an interaction between *RpMR1* and *TLR4* in the immune response. This study provides the first functional evidence of mannose receptor-mediated immunity in mollusks, offering new insights into the molecular defense mechanisms of *R. philippinarum* against bacterial infection.

**Supplementary Information:**

The online version contains supplementary material available at 10.1007/s44307-025-00075-7.

## Introduction

Mollusks, like many metazoans, rely on conserved immune strategies, including cellular and humoral responses, to combat pathogens (Tiscar et al. 2004). Collectively, cellular and humoral immunity enable mollusks to mount effective defense responses against environmental stressors, facilitating phagocytosis of foreign particles and detoxification of harmful substances (Soares-da-Silva et al. 2002). While mollusks possess a well-developed immune system, their defense mechanisms differ fundamentally from vertebrate immunity. Unlike vertebrates, which employ both adaptive and innate immune responses, mollusks rely exclusively on innate immunity—including enhanced phagocytic activity, pathogen recognition receptors, and effector molecules—to combat pathogenic invasion (Hoffmann et al. 1999; Little et al. 2005). Shellfish are widely recognized as susceptible to infection by various bacterial species during their growth, including *V. tasmaniensis*, *V. crassostreae*, *V. parahaemolyticus*, and *V. anguillarum.* Numerous studies have investigated the molecular mechanisms underlying shellfish resistance to these pathogens, focusing primarily on molecular genetic processes (Islam et al. 2022; Namwong et al. 2022)*.* Accordingly, our previous transcriptomic studies on *Ruditapes philippinarum* subjected to *Vibrio anguillarum* stress have identified toll-like receptor (*TLR*) and *MR* as potential candidate genes for screening disease resistance in clams (Yin et al. 2022a, 2022b). Indeed, pattern recognition receptors (PRRs), including *TLRs* and mannose receptors (*MRs*), function as molecular sensors for the specific recognition of pathogens and play a pivotal role in the elimination of invading microorganisms (Li et al. 2020a; Ni et al. 2007).

The MR is a type of pattern recognition receptor (PRR) that functions as a "non-standard" PRR, primarily contributing to host defense by recognizing and binding to endogenous ligands or pathogen-associated molecules. Through this interaction, *MR* plays a key role in modulating the immune response and maintaining immune homeostasis under external stress (Gazi et al. 2009; Huo et al. 2024b; Yin et al. 2022b). The immunological relevance of *MR*-mediated responses has been highlighted in studies reporting antiviral and antibacterial activities against Vibrio species in aquatic animals such as *Procambarus clarkii* and *Epinephelus coioides* (Man et al. 2018; Zhang et al. 2021). As a member of the *MR* family within the C-type lectin receptor (CLR) superfamily, *MR* is classified as a type I transmembrane protein (Stahl et al. 1998). Structurally, it comprises an extracellular cysteine-rich (CR) domain, a fibronectin type II (FN II) domain, and eight tandem C-type lectin-like domains (CTLDs), along with a transmembrane segment and a short cytoplasmic tail (Gazi and Martinez-Pomares 2009). Functional studies have demonstrated a synergistic interaction between the FN II and CTLD2 domains, which enhances the uptake of glycosylated collagen. Additionally, CTLD4 has been identified as a critical domain involved in modulating T cell cytotoxicity (Martinez-Pomares 2012; Schuette et al. 2016). The MR protein also possesses ligand-binding capabilities that enable it to transduce signals from the extracellular environment into the cytoplasm. Its CTLDs can specifically recognize and bind carbohydrate ligands such as mannan, trehalose, and N-acetylglucosamine on various endogenous and exogenous molecules, subsequently initiating immune responses (Zhang et al. 2021). In *Megalobrama amblycephala* (bream), it has been shown that MR specifically binds to chitosan oligosaccharide (COS) and mediates COS6 uptake by macrophages via lectin-dependent endocytosis. This interaction modulates the expression of tumor necrosis factor receptor-associated factor (*TRAF*), interleukins (*ILs*), and nitric oxide synthase (NOS), underscoring the pivotal role of *MR* in ligand recognition, internalization, and immune regulation (Ouyang et al. 2021).

*R. philippinarum* commonly known as the Manila clam, belongs to the phylum Mollusca, class *Bivalvia*, order Eulamellibranchia, family Veneridae, and genus *Ruditapes* (Li et al. 2019). As an ecologically and economically important species, *R. philippinarum* exhibits several advantageous traits, including broad salinity and temperature tolerance, resistance to pollution, and rapid growth (Yin et al. 2022b). However, in recent years, the aquaculture industry of *R. philippinarum* has faced increasing threats due to bacterial infections, protozoan parasites, and environmental stressors (Le Roux et al. 2016; Nie et al. 2016; Waki et al. 2012). Among these, vibriosis—caused by the Gram-negative pathogen *Vibrio anguillarum*—is particularly detrimental, leading to hemorrhagic septicemia, high mortality in farmed clams, and significant economic losses (Ren et al. 2021).

Understanding the immune defense mechanisms of *R. philippinarum* against bacterial pathogens is essential for the development of genetically improved, disease-resistant strains. Transcriptomic analyses have provided key insights into the clam’s immune responses. For instance, studies investigating the immune response to *V. parahaemolyticus* infection have highlighted the critical role of the *C1q* gene in disease resistance(Yu et al. 2023b). Other transcriptomic investigations have demonstrated that exposure to various pathogen-associated molecular patterns (PAMPs) triggers the activation of immune effectors such as apextrin, along with downstream activation of the NF-κB signaling pathway (Jiang et al. 2020b, 2022). Our previous comparative transcriptomic study revealed the involvement of *MR* in the phagosome pathway, suggesting a significant role for *MR* in Vibrio tolerance in *R. philippinarum* (Yin et al. 2022a, b). Further supporting this, evidence from *TLR* studies underscores a cooperative interaction between *MR* and *TLR4* in pathogen recognition, where crosstalk between PRRs enhances both innate and adaptive immune responses(Loures et al. 2015). Additionally, mannose-binding lectin (MBL)—another member of the C-type lectin receptor (CLR) family—has been shown to potentiate *TLR4* signaling through direct interaction with its leucine-rich repeat (LRR) domain (Wang et al. 2011). Although *MR* and *MBL* differ in ligand specificity, their shared classification within the CLR family suggests conserved immunomodulatory functions in host defense.

In the present study, we identified and characterized the expression patterns of mannose receptor (*MR*) genes in *R. philippinarum* following *V. anguillarum* challenge, revealing a significant role for *MR* in the host immune response. In vitro antibacterial assays demonstrated that the recombinant MR protein (RpMR1) exerted inhibitory effects against three Gram-negative bacterial strains. Furthermore, in vivo overexpression of RpMR1 via injection significantly increased the survival rate of *R. philippinarum* under *V. anguillarum* infection, underscoring its protective function. Conversely, silencing of the *RpMR1* gene through RNA interference led to a marked downregulation of *TLR4* expression. This finding suggests that *MR* may activate the *TLR* signaling pathway during pathogen invasion, potentially interacting with *TLR4* to facilitate phagocytosis and coordinate innate immune responses. Together, these data support a synergistic role for *MR* and *TLR4* in mediating host defense against bacterial infection. Overall, this study provides novel insights into the immune mechanisms of *R. philippinarum* and highlights *MR* as a potential target for enhancing disease resistance in molluscan aquaculture.

## Materials and methods

The clam genome data used in this study were obtained from our previous work (Yan et al., 2019), with the NCBI biological project number PRJNA479743(Yan et al. 2019). Candidate *MR* genes were screened using Hidden Markov Models (HMMs) (Jiang et al. 2021). The putative isoelectric point (PI) and molecular weight of the proteins were calculated using the Compute pI/Mw tool (http://web.expasy.org/compute_pi/, http://web.expasy.org/protparam/) (Jiang et al. 2020a). Genome sequences of *MR* genes from other organisms were downloaded, with relevant database identifiers provided in Table S1. Multiple sequence alignments of *RpMR* genes were performed using DNAMAN software (https://www.lynnon.com/dnaman.html). Phylogenetic analysis was conducted using MEGA 7.0 (http://www.megasoftware.net) employing the maximum likelihood (ML) method, followed by 1000 bootstrap replicates for tree construction (Bai et al. 2020).

### RpMR gene structure, chromosome location and evolution analysis

The gene structure of *RpMR* was analyzed using the Gene Structure Display Server (GSDS) 2.0 (http://gsds.gao-lab.org/index.php), which allowed for the visualization of the gene’s coding sequence (exons), untranslated regions (UTRs), and introns, derived from the gene annotation file(Hu et al. 2015; Jiang et al. 2021; Yin et al. 2022a). Functional and evolutionary analysis of characterized sequences was performed using the MEME web server, which provides a comprehensive platform for sequence motif discovery and analysis (Bailey et al. 2009; Jiang et al. 2021; Yin et al. 2022a). Chromosomal location data for *RpMR* genes were retrieved from the *R. philippinarum* genome annotation file(Jiang et al. 2021; Wang et al. 2020; Yin et al. 2022a). The chromosomal positions and relative distances of *RpMR* genes were visualized using MG2C 1.1 (http://mg2c.iask.in/mg2c_v1.1/).

### Infection experiment of *V. anguillarum* in *R. philippinarum*

Infection experiments were conducted at various time points following *V. anguillarum* exposure using wild clam populations collected from Jinshitan, Dalian City, Liaoning Province. The clams (shell length: 30.8 ± 0.3 mm, shell width: 12.4 ± 0.3 mm, shell height: 20.1 ± 0.4 mm, total weight: 7.4 ± 0.4 g) were acclimatized in tanks containing seawater (water temperature: 21 ± 0.3 °C, pH: 8.2 ± 0.1, salinity: 32 ± 0.2 ppt). The experimental system consisted of nine 30-L aquaria, arranged in triplicate groups (three tanks per treatment), each housing 70 *R. philippinarum* specimens. During the 7-day acclimatization phase, the clams were reared in clean seawater and fed daily with Chlorella vulgaris. For the challenge experiment, clams were immersed in *V. anguillarum* at a concentration of 1 × 10⁷ CFU/mL. Samples were collected at 0-, 12-, 24-, 48-, 72-, and 96-h post-challenge, with each time point sampled in triplicates. Hepatopancreas tissues were harvested, snap-frozen in liquid nitrogen, and stored at −80°C for subsequent RNA extraction.

### Collection of samples from different tissues and different developmental stages

Wild clams (*Ruditapes philippinarum*) were collected from Jinshitan, Dalian, Liaoning Province. Tissue samples from adults included adductor muscle (AM), mantle (M), foot (F), gill (GI), pipe (P), and digestive gland (DG). Developmental stages analyzed were embryos and larvae, categorized as fertilized egg (FE), first polar body (PB1), second polar body (PB2), 2-cell (TC), 8-cell (EC), blastula (B), gastrula (G), trochophore larvae (T), D-larvae (D), Umbo larvae (U), pediveliger larvae (P), single siphon spat (S) and juveniles (J). The expression of *MR* gene (*MR1*-*MR6*) was assessed across tissues and developmental stages by qPCR and RNA-seq analysis, respectively. The expression characteristics of *MR* genes were performed based on transcriptome sequencing data at different developmental stages of *R. philippinarum* (Zhang et al. 2022).

### Extraction of RNA and synthesis of cDNA from *R. philippinarum*

Three biological replicates of clam samples were analyzed per experimental group. Total RNA was extracted using the phenol–chloroform method (Yu et al. 2023b; Zhang et al. 2022). TransScript ® IIOne Step gDNA Removal and cDNA Synthesis SuperMix (Beijing, all gold) reverse transcription kit is used to synthesize cDNA. The reverse transcription reaction (20 μL total volume) contained the following components: 500 ng of total RNA, use 0.5 μL of anchored Oligo (dT) 20 Primer (0.5 μg/μL), 0.5 μL of random primer (N9) (0.1 μg/μL), 10 μL of 2 × TSII Rection Mix, 1 μL of TranScript IIRT/RI Enzyme Mix, 1 μL of gDNA remover, RNase-free water was added to adjust the final volume to 20 μL. The reaction mixture was incubated at 50 °C for 15 min and heat at 85 °C for 5 s, and the resulting cDNA was stored at −20° C.

### qPCR analysis

Fluorescent quantitative primers were designed using Primer 5 software (Premier Biosoft International). Primer sequences are listed in Table S2 (Li et al. 2019, 2020b). The β-actin was used as the internal reference gene (Yin et al. 2022b). Real -time quantitative PCR uses TB Green Premix Extaqii (TAKARA, Tokyo, Japan) (Jiang et al. 2022). The total reaction volume was 20 μL, comprising 2 μL of cDNA (50 ng/μL), 1 μL of positive and negative primers, 10 μL of TB Green PCR Master Mix, and 6 μL of H₂O. The amplification conditions were as follows: initial denaturation at 95 °C for 3 min, followed by 40 cycles of denaturation at 94 °C for 30 s, annealing at 60 °C for 30 s, and extension at 72 °C for 30 s. Relative gene expression levels were analyzed using the 2^^−ΔΔCT^ method (Schmittgen et al. 2008). Statistical analysis of the data was performed using SPSS 20.0. Single-factor analysis of variance (ANOVA) followed by the Tukey's test was used to assess significant differences between the groups. A *p*-value of less than 0.05 (*P* < 0.05) was considered statistically significant.

### RpMR1 prokaryotic expression technology

The prokaryotic expression of the *RpMR1* gene was carried out at Shanghai Sangon Bioengineering Co., Ltd. The coding region of the gene was amplified by PCR to obtain a sufficient quantity of the product, which was then inserted into the PET-28A (+) vector using restriction enzymes. The recombinant plasmid was introduced into E. coli Rosetta (DE3) cells through transformation. The transformed cells were plated on LB agar containing 30 µg/mL kanamycin and incubated at 37 °C. A single colony was selected and cultured in liquid medium with kanamycin at 37 °C. Once the OD600 reached 0.6, protein expression was induced by adding 0.5 mM IPTG, and the cells were incubated for an additional 6 h. After harvesting, the cells were resuspended in 1 × PBS (pH 7.4) and subjected to ultrasonic disruption. The suspension was centrifuged to separate the supernatant and pellet, and the pellet was resuspended in 50 mM Tris, 300 mM NaCl, and pH 8.0. The protein was solubilized in a buffer containing 8 M urea, 50 mM Tris, 300 mM NaCl, and 0.1% Triton X-100 (pH 8.0). After ultrasonic treatment, the mixture was centrifuged to obtain the supernatant. Protein purification was achieved through dialysis against increasing concentrations of urea in Tris-NaCl buffer. Protein expression and purity were confirmed by SDS-PAGE using a 12% separation gel and 5% stacking gel, with molecular weight estimation by comparison to protein standards. The purified protein was concentrated using PEG20000 after dialysis, filtered through a 0.45 µm filter, and stored at −80°C for future use (1:1000, Sangon Biotech, China).

### Injection of RpMR1 recombinant protein experiment and NOS detection

Wild clams from Jinshi Beach in Dalian were used for *V. anguillarum* injection experiments. The clams were randomly assigned to four groups: A (PBS + MR), B (Va + MR), C (Va + PBS), and D (PBS control group). The injection volumes of *V. anguillarum*, recombinant protein, and PBS were 50 μL each, with concentrations of 1 × 10⁷ CFU/mL for *V. anguillarum*, 200 μg/mL for the recombinant protein, and PBS as the control. Mortality rates were recorded at 0, 6, 12, 24, 48, 72, and 96 h post-injection. Hepatopancreas tissues were collected, immediately frozen in liquid nitrogen, and stored at −80°C. Nitric oxide (NO) activity was measured using the nitric oxide assay kit (Nanjing Jiancheng, China) following the manufacturer's instructions.

### RpMR1 protein bacteriostatic experiment

The recombinant RpMR1 protein was tested for bacteriostatic activity against eight different bacterial strains, including *B. subtilis*, *S. aureus*, *V. anguillarum*, *V. parahemolyticus*, *V. harveyi*, *V. splendidus*, *V. algnolytiacus* and *E. coli.* After the bacteria were activated for a second time, a coating experiment was performed, followed by inoculation of each bacterial strain into 10 mL of LB broth. The recombinant protein was added to a final concentration of 20 μg/mL. The cultures were incubated for 10 h under constant shaking conditions (37°C, 200 rpm). During the experiment, bacterial growth was monitored by measuring the optical density at 600 nm (OD600) every 2 h (Jiang et al. 2022).

### RNA silencing

For the dsRNA injection experiments, wild clams collected from Jinshi Beach in Dalian were used. The clams were divided into four groups: experimental group (RNAi), negative control group (pEGFP), positive control group (DEPC), and blank control group (Con), with three clams in each group. In the experimental group, 25 μg/100 μL of synthetic dsRNA RpMR1 interference strand was injected. The negative control group received 25 μg/100 μL of dsRNA-pEGFP-N3 interference strand (purchased from Dalian Jima Company, China) (Yu et al. 2023a). The positive control group was injected with 100 μL of DEPC, while the blank control group received no treatment. After 36 h post-injection, hepatopancreas tissues were collected, snap-frozen, and stored at −80°C for subsequent analysis.

### Ethic statement

All animal procedures in this experiment were conducted in accordance with the ethical guidelines set by the Experimental Animal Center of Dalian Ocean University to minimize animal suffering. The study adhered to national laws and regulations governing the use of experimental animals.

## Results

### Identification of RpMR gene family

In this study, a total of 13 mannose receptor (*MR*) genes, designated *RpMR1* to *RpMR13*, were identified in the *R. philippinarum* genome (NCBI BioProject: PRJNA479743). These genes were identified based on the presence of C-type lectin-like domains (CTLDs) using the HMMER search with the Pfam model PF00059.23. The detailed characteristics of the identified *RpMR* genes are summarized in Table 1. The predicted RpMR proteins exhibit a wide range of molecular weights, from 11.27 kDa to 320.37 kDa, and theoretical isoelectric points (pI) ranging between 4.42 and 6.82, suggesting functional and structural diversity among the MR family members in *R. philippinarum*.

**Table 1:** The detail information of *MR* gene family members in *R. philippinarum* genome

Gene name	Gene ID	Chr number	Chromosome length	Chromosomal location	Gene stand	Molecular weight (kDa)	PI
*RpMR1*	xfSc0000262.8	10	41,560,981	33,350,163–33378259	-	311.11	4.85
*RpMR2*	xfSc0000020.9	10	41,560,981	10,863,413–10884496	+	131.82	8.35
*RpMR3*	xfSc0001192.5	4	56,440,294	18,273,111–18,310,054	+	70.59	5.01
*RpMR4*	xfSc0000495.6	10	41,560,981	17,403,407–17415810	-	83.33	4.85
*RpMR5*	xfSc0005707.1	0	-	-	-	62.14	4.54
*RpMR6*	Sc0000045.9	9	37,028,854	35,020,290–35027760	+	53.11	5.04
*RpMR7*	xfSc0000495.7	10	41,560,981	17,418,034–17480613	-	320.37	5.19
*RpMR8*	xfSc0000519.4	15	50,027,941	35,425,935–35,455,941	+	142.81	6.37
*RpMR9*	xfSc0000519.5	15	50,027,941	35,409,151–35424993	+	74.43	6.82
*RpMR10*	xfSc0000108.30	12	33,673,787	23,660,287–23663732	+	31.47	6.74
*RpMR11*	xfSc0001787.6	1	58,439,140	3,112,136–3,113,018	+	18.17	5.75
*RpMR12*	xfSc0002266.5	0	-	-	+	51.71	5.59
*RpMR13*	xfSc0003897.1	0	-	-	+	11.27	4.42

### RpMR gene family structure, conserved motif analysis and chromosome location

The 13 identified *RpMR* genes exhibited considerable structural diversity, with exon numbers ranging from 2 to 60. Phylogenetic analysis and intron–exon structure comparisons are presented in Figure S1, illustrating evolutionary relationships among the *RpMR* family members. Conserved motif analysis revealed 10 distinct motifs (designated motifs 1–10) across the RpMR proteins (Fig. S2), while domain architecture analysis identified seven major structural domains: C-type lectin domain (CLECT), tetratricopeptide repeat (TR), coagulation factor 5/8 C-terminal domain (FA58C), fibrinogen C-terminal domain (FTP), kringle domain (KR), signal peptide, and transmembrane region (Fig. S3). All RpMR proteins contained at least one CLECT domain, with copy numbers ranging from 1 to 14 per protein, indicating potential functional redundancy or diversification. Multiple sequence alignment of *RpMR1–RpMR13* highlighted several conserved amino acid residues, including cysteine residues, Ca^2^⁺-binding sites, acidic amino acids, and aromatic residues such as phenylalanine and tryptophan (Fig. S4), which are characteristic features of C-type lectin family proteins. Chromosomal localization analysis revealed that *RpMR* genes are distributed across several chromosomes, with 10 genes mapped to six annotated chromosomes. Notably, chromosome 10 harbors three *RpMR* genes, indicating a possible gene cluster (Fig. S5). The remaining three genes—*RpMR5*, *RpMR12*, and *RpMR13*—were located on unplaced scaffolds. Comprehensive annotations, including chromosome lengths and genomic coordinates, are provided in Table 1.

### Phylogenetic analysis of the RpMR gene family

To investigate the evolutionary relationships of the *RpMR* gene family, a phylogenetic tree was constructed using 142 *MR* amino acid sequences from nine representative species, selected based on the presence of the C-type lectin-like domain (HMM model PF00059.23) (Fig. S6). The phylogenetic analysis revealed that *MR* genes cluster into two major branches. Notably, *MR* sequences from molluscan shellfish, including *R. philippinarum*, *Crassostrea virginica*, *C. gigas*, and *Biomphalaria glabrata*, grouped predominantly within a single large clade. These results suggest that the *MR* genes in mollusks have undergone evolutionary conservation, reflecting shared ancestry and potentially conserved functional roles across molluscan species.

### Expression pattern of RpMR gene at different time points after *V. anguillarum* stress

**Fig. 1:**
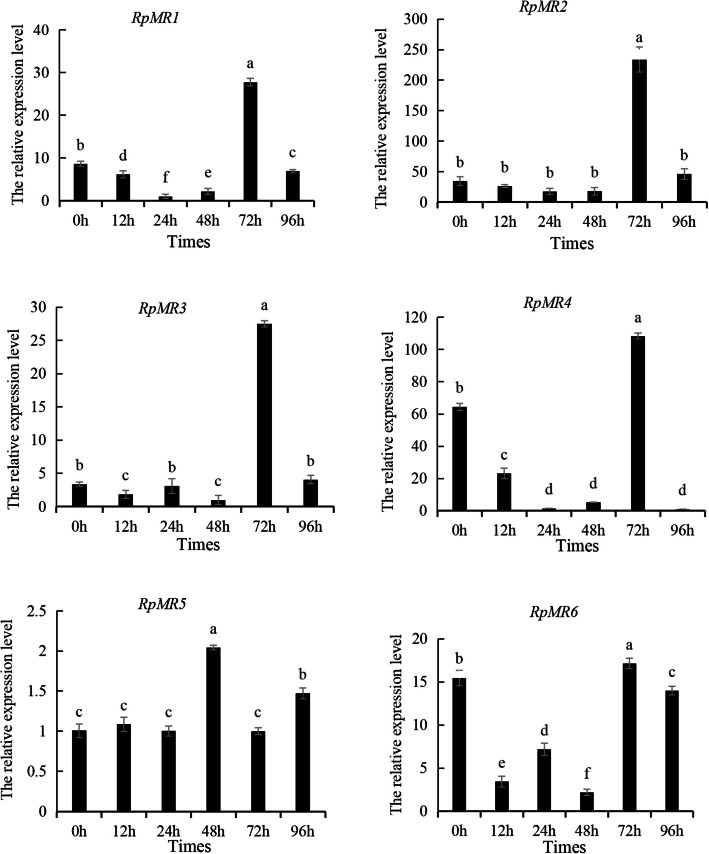
Expression of *RpMR* genes at different time points after *V. anguillarum* stress. Note: Different letters indicate that there are significant differences in gene expression at different time points (*P* < 0.05)

The expression pattern of *RpMR* gene at different time points after *V. anguillarum* challenge was shown in Fig. 1. The results showed that *RpMR* expression was greatly increased following *V. anguillarum* challenge. Notably, the expression levels of all *RpMR1*, *RpMR2*, *RpMR3*, *RpMR4*, and *RpMR6* peaked at 72 h post-infection, reaching 3.2-, 6.8-, 8.3-, 1.68-, and 1.1-fold increases, respectively, relative to the baseline expression at 0 h. These findings suggest a time-dependent transcriptional regulation of *RpMR* genes, particularly during the late phase of the immune response to *V. anguillarum*.

### Expression pattern of *RpMR* gene in different tissues

**Fig. 2:**
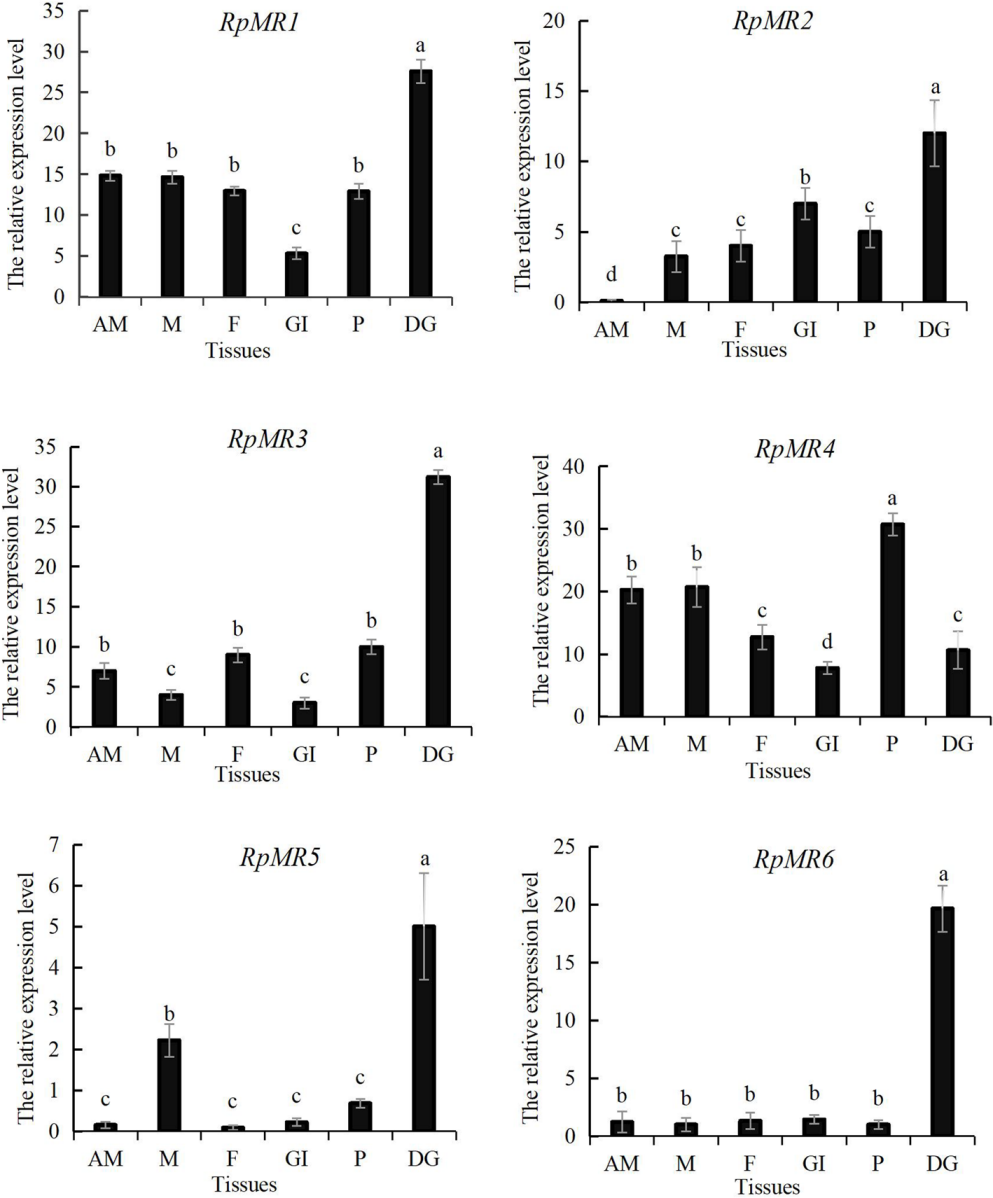
The expression of the *RpMR* gene in different tissues of Manila clam. Note: Different letters indicate that genes are significantly different in different tissues (*P* < 0.05). Adductor muscle (AM), mantle (M), foot (F), gill (GI), pipe (P), digestive gland (DG)

The expression of *RpMR* gene in different tissues of clam was examined by qPCR (Fig. 2). Results showed that *RpMR* was expressed in adductor muscle, mantle, foot, gill, siphon, and digestive gland, which mainly expressed in the hepatopancreas. The expression of *RpMR1* was significantly higher in hepatopancreas tissue (twofold), compared with adductor muscle, mantle, foot and gill tissue (*P* < 0.05). The expression level of *RpMR2* gene was the highest in the hepatopancreas, followed by the gill tissue, and the expression level in the adductor muscle was the lowest. The expression of *RpMR2* was significantly higher in hepatopancreas tissue (12-fold), compared with adductor muscle tissue (*P* < 0.05). The expression level of *RpMR3* gene was the highest in the hepatopancreas and the lowest in the gills. The tissue-specific expression analysis revealed that *RpMR3* was significantly upregulated in the hepatopancreas, with expression levels exceeding a threefold increase compared to other tissues (*P* < 0.05). Similarly, *RpMR4* and *RpMR5* also exhibited their highest expression levels in the hepatopancreatic tissue, suggesting that this organ may serve as a key site for mannose receptor-mediated immune responses in *R. philippinarum*.

### Detection of differentially expressed of TLR pathway genes under stress of *V. anguillarum*

**Fig. 3:**
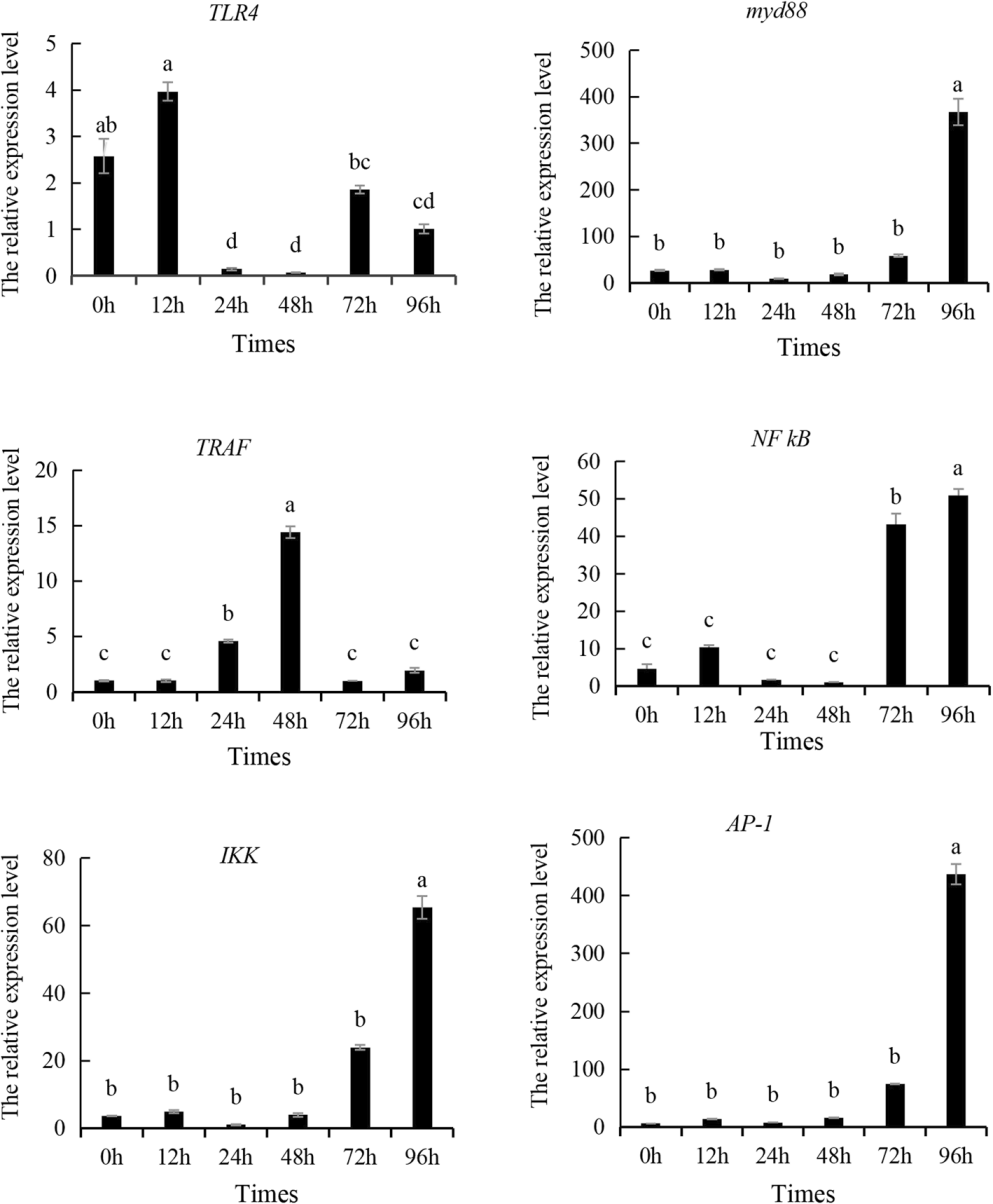
Expression of Toll-like receptor pathway related genes in *V. anguillarum* under stress. Note: Different letters indicate that there are significant differences in gene expression at different time points (*P* < 0.05)

The expression levels of genes related to TLR pathway had different expression characteristics after *V. anguillarum* stress. As shown in Fig. 3, the expression of the *TLR* gene in response to *V. anguillarum* stress exhibited a dynamic pattern. Expression levels significantly increased at 12 h post-infection, decreased at 24 and 48 h, and rose again at 72 h. Furthermore, most genes within the TLR signaling pathway were activated during the infection. Notably, the downstream signaling components to the TLRs-including myeloid differentiation primary response gene 88 (*MyD88*), nuclear factor kappa-B (*NF-κB*), IκB kinase (*IKK*), activator protein-1 (*AP-1*), and tumor necrosis factor receptor-associated factor 6 (*TRAF6*)—were significantly upregulated, highlighting the involvement of the *TLR* pathway in orchestrating immune responses to *V. anguillarum*.

### Expression patterns of *RpMR* genes in different developmental stages

The *RpMR* gene expression at different developmental stages were plotted into a heat map (Fig. S7). The results showed that all *RpMR* genes expressed with the growth and development of the clam, and the expression of most *RpMR* genes increased significantly in the D larva. The expression of the *RpMR1* gene in the pediveliger larvae stage was decreased compared to the Umbo-larvae stage, but higher than other *RpMR* genes in the Umbo-larvae stage.

### RpMR1 recombinant protein by prokaryotic expression

**Fig. 4:**
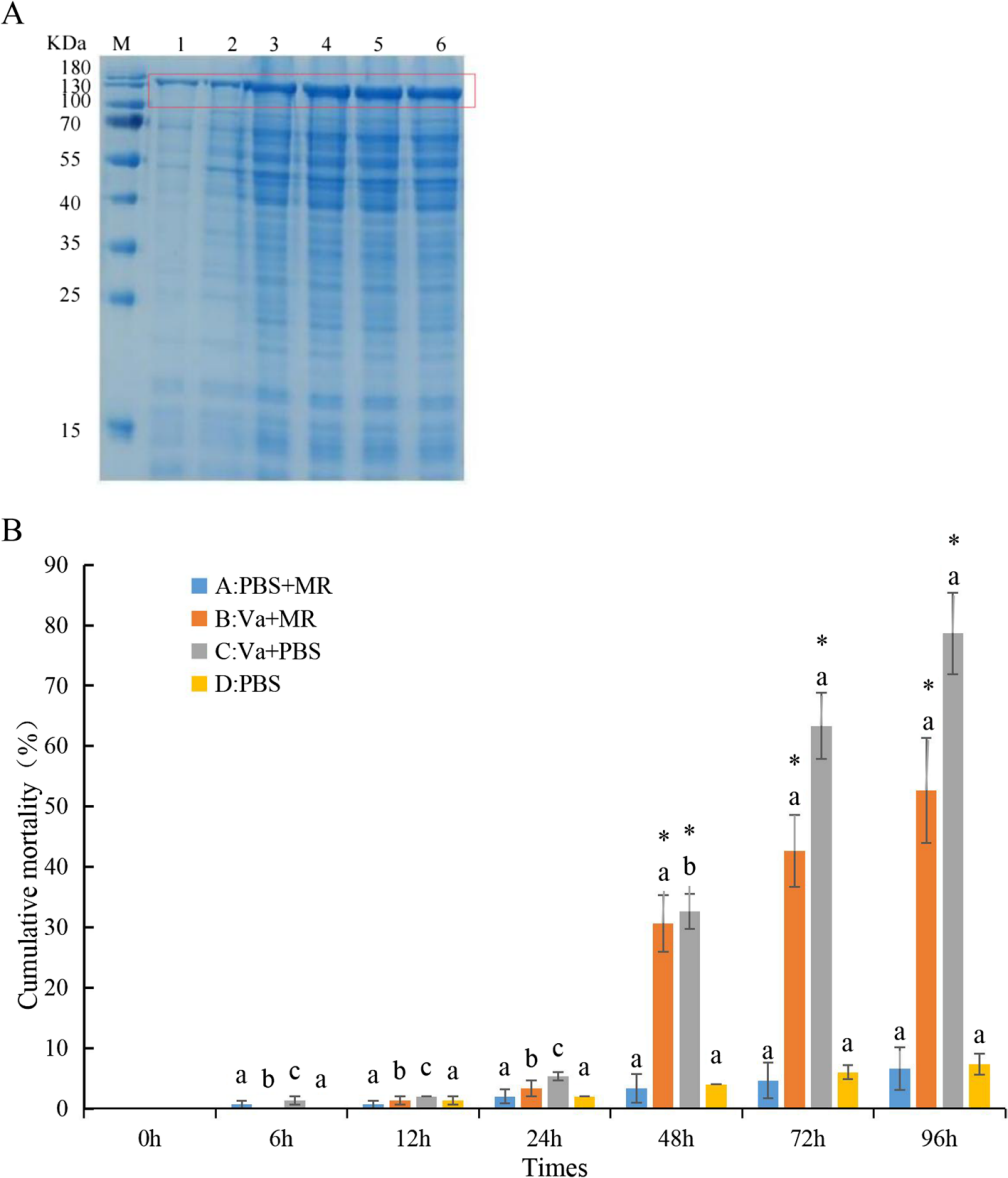
SDS-PAGE electrophoresis of RpMR1 recombinant protein. (**A**) Note: M: Protein Marker; 1: uninduced sample; 2-6: induced samples. (**B**) The effect of RpMR1 on the survival rate of *R. philippinarum* infected by *V. anguillarum*. Note: A: PBS + RpMR, B: *V. anguillarum* + RpMR, C: *V. anguillarum* + PBS and D: PBS. Use letters to indicate significant differences at different time points, with an asterisk indicating differences between different groups at the same time point (*P* < 0.05)

RpMR1 recombinant protein was successfully expressed in PET-28a (+) (Fig. S8). The purified RpMR1 recombinant protein was obtained with a concentration of (0.5 mg/mL). After purification, a band appeared at the corresponding position of theoretical 123 kDa by SDS-PAGE electrophoresis analysis, indicating that the protein was successfully purified (Fig. 4A). Western blot analysis confirmed the successful expression of the target protein (Fig. S9).

### Survival rate of clam after *V. anguillarum* infection with injection of RpMR1 recombinant protein

To confirmed the function of recombinant protein RpMR1, survival rate of clam after *V. anguillarum* infection with injection of RpMR1 was performed. Each experimental group had 150 clams, divided equally into 3 groups, with 50 clams in each group. As shown in Fig. 4, the cumulative mortality rate of group C (*V. anguillarum* + PBS) was 78.6% at the 96 h of the experiment, and the cumulative mortality rate of group B (*V. anguillarum* + MR) was 52.7%. Group C has a cumulative mortality rate of 15.9% higher than Group B. *R. philippinarum* in group A (PBS + MR) and group D (PBS) showed the same mortality rate (Fig. 4B). It indicated that the RpMR1 recombinant protein may enhance the ability of clams to resist *V. anguillarum.*

### Effects of RpMR1 protein injection on TLR pathway gene expression and nitric oxide synthase activity in *R. philippinarum*

**Fig. 5:**
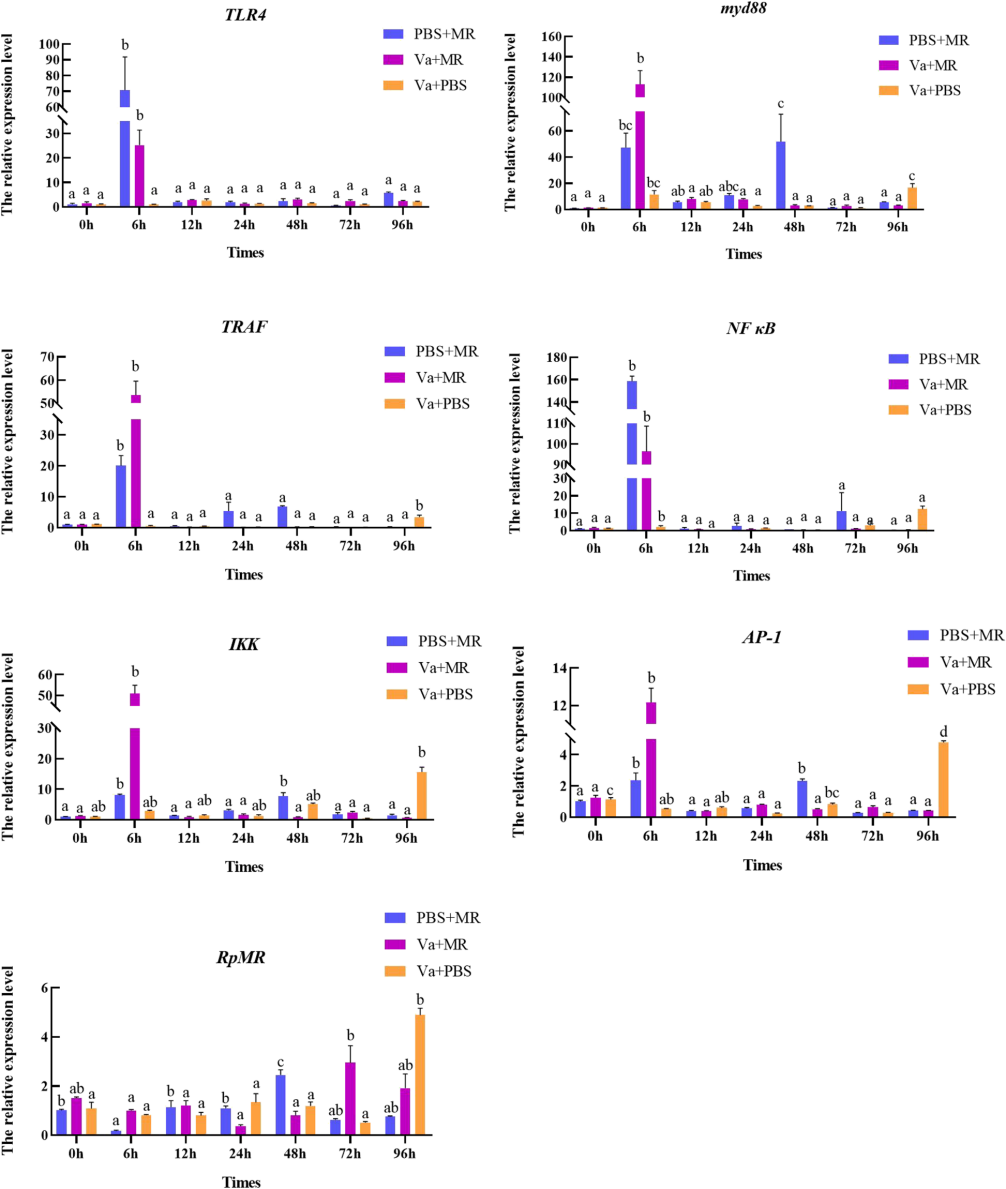
Expression of Toll-like receptor pathway related genes after injection of RpMR1 recombinant protein. Note: Different lowercase letters indicate that there are significant differences in gene expression at different time points (*P* < 0.05)

**Fig. 6:**
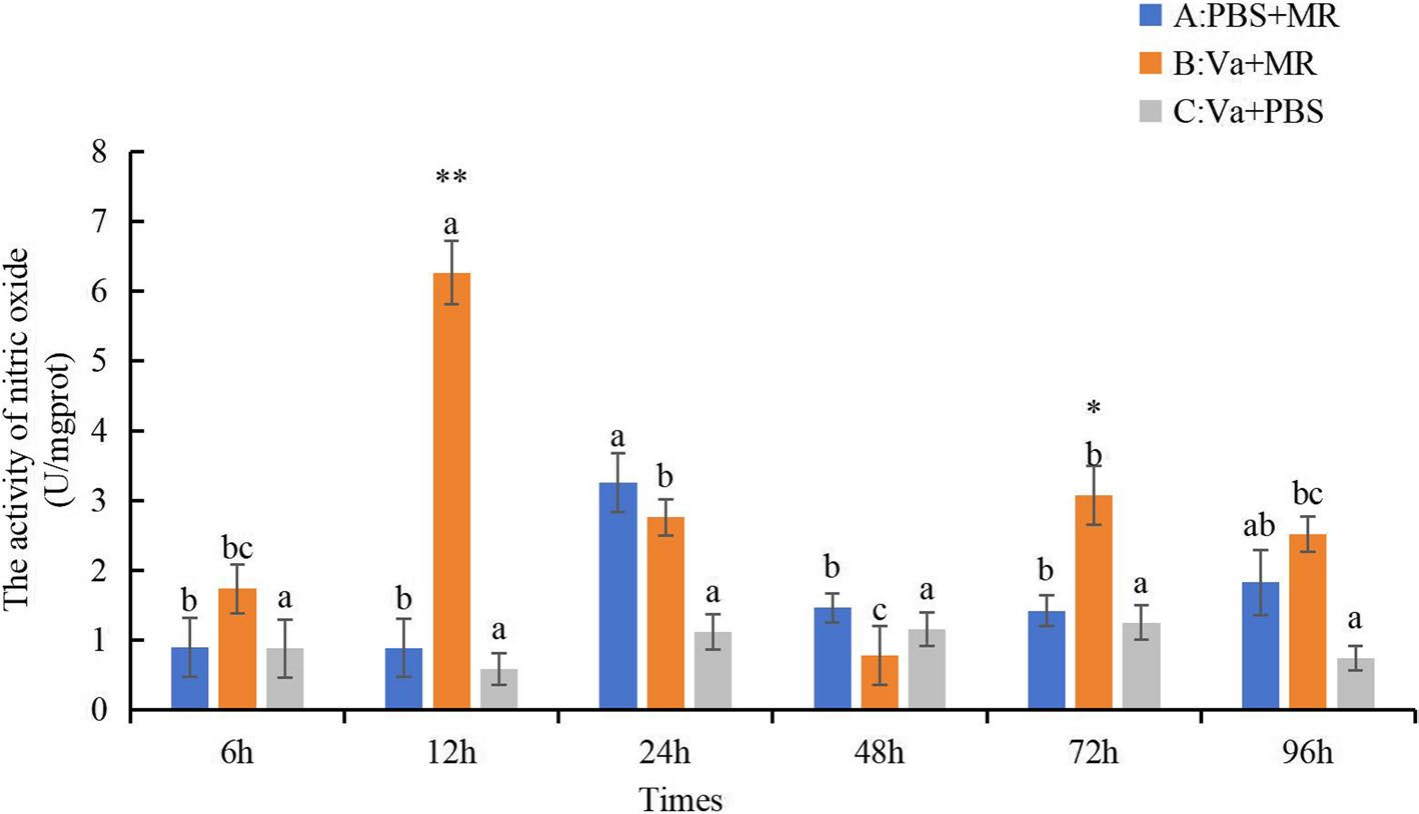
NOS activity after injection of RpMR1 recombinant protein. Note: Different letters indicate the difference of the same treatment at different time points (*p* < 0.05)."*"indicates extremely significant difference group at the same time point (*p* < 0.01)

Expression of TLR pathway-related genes after injection of RpMR1 recombinant protein was shown in Fig. 5. The results showed that the gene expressions of *TLR*, *MyD88*, *TRAF*, *NF-κB*, *IKK*, and *AP-1* in the (Va + MR) group peaked at 6 h of post infection (*P* < 0.05). The expression of *TLR*, *TRAF*, and *NF-κB* in the (PBS + MR) group peaked at 6 h and the amount reached the peak, and *MyD88*, *IKK*, *AP-1* reached the highest peak at 48 h. Most TLR pathway genes in (Va + PBS) group peaked at 96h. The nitric oxide synthase NOS activity of (Va + MR) group was significantly increased at 12 h and 72 h (*P* < 0.05), and was significantly higher than that of (PBS + MR) group and (Va + PBS) group (*P* < 0.01) (Fig. 6).

### RpMR1 protein antibacterial activity

**Fig. 7:**
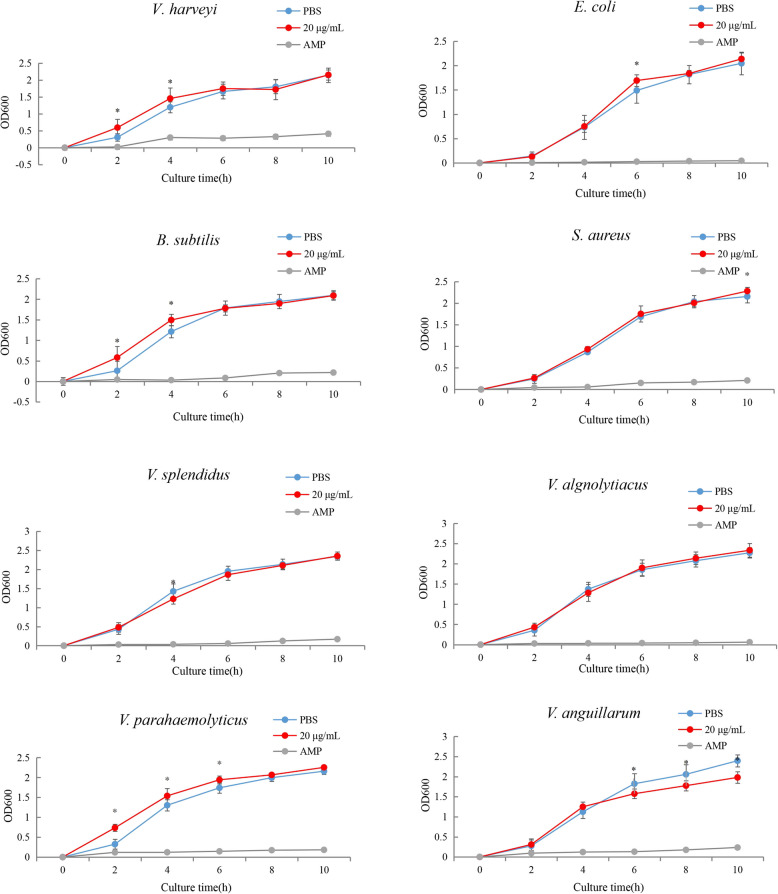
The inhibition curve of RpMR1 protein on the growth of different bacteria was determined using. Note: Ampicillin as a positive control and PBS as a negative control"*"*p* < 0.05

In vitro antibacterial activity assays of the *Ruditapes philippinarum* recombinant protein RpMR1 were conducted (Fig. 7). The RpMR1 protein exhibited significant inhibitory effects against several Gram-negative bacteria, including *Vibrio anguillarum*, *V. splendidus*, and *V. alginolyticus*. Significant growth inhibition was observed against *V. splendidus* at 4 h (*P* < 0.05), with sustained suppression of *V. anguillarum* at 6 h, 8 h, and 10 h (*P* < 0.05). However, no inhibitory activity was detected against *Bacillus subtilis*, *Staphylococcus aureus*, *Vibrio parahaemolyticus*, *V. harveyi*, or *Escherichia coli*.

### Changes of *RpMR1* and TLR pathway gene expression levels in different

**Fig. 8:**
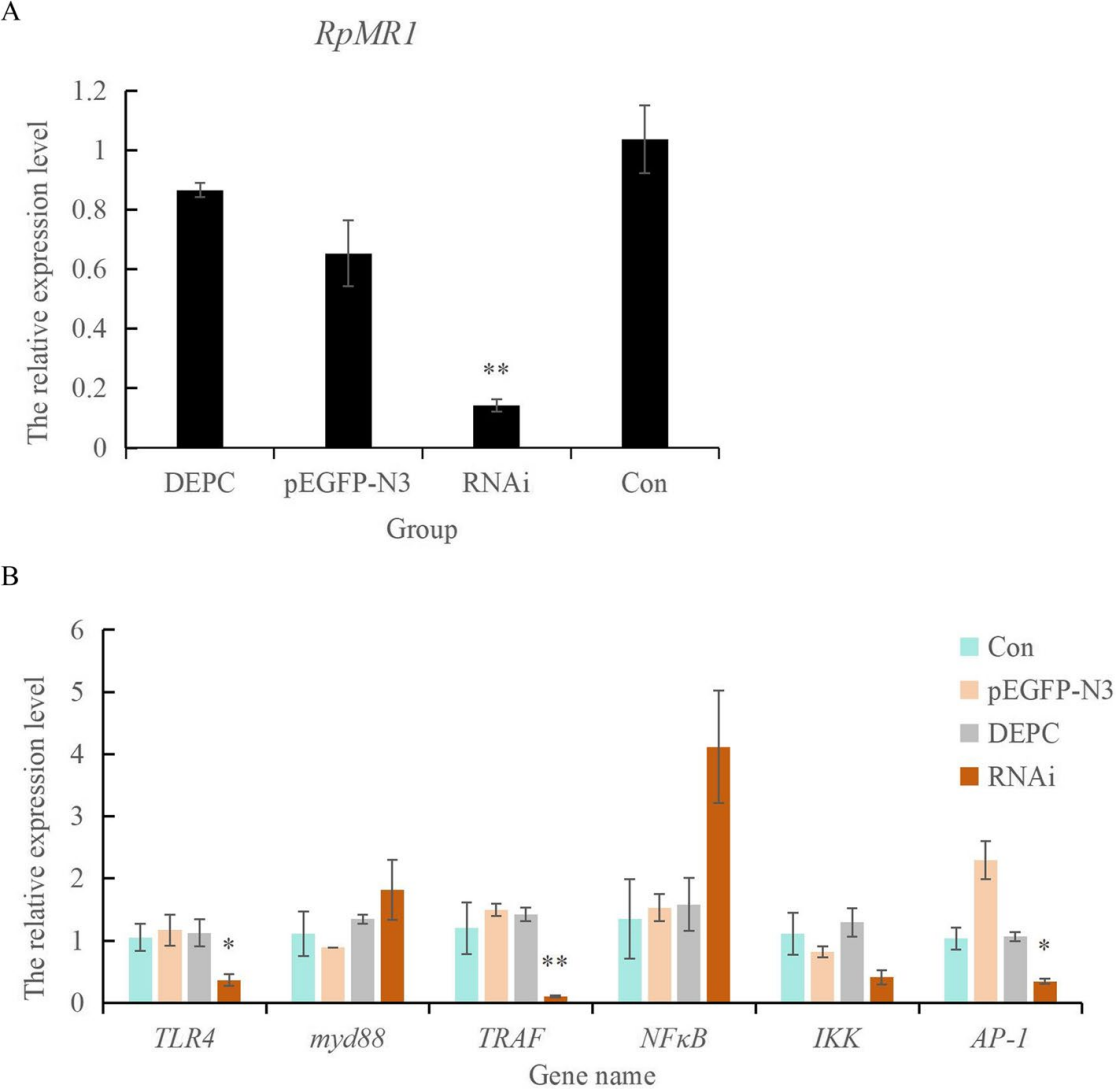
Changes of gene expression levels in different experimental groups by injecting RNA interference probes. **A** is the change of *MR* expression, **B** is the change of TLR pathway gene expression. Note: The RNAi group was injected with 25 μg/100 μl of synthetic dsRNA *RpMR* interference strand, and the negative control group was injected with 25 μg/100 μl of dsRNA-pEGFP-N3 interference strand. The positive control group was injected with 100 μl of DEPC. Con is no treatment control group."*"indicate significant differences between genes after MR interference and the control group (*p* < 0.05),"**"(*p* < 0.01)

Following RNA interference (RNAi) targeting the RpMR1 gene, its expression was significantly reduced (*P* < 0.01). Concurrently, key genes in the TLR signaling pathway were also downregulated: *TRAF6* expression decreased significantly (*P* < 0.01), while *TLR4* and *AP-1* expression levels were significantly reduced (*P* < 0.05) (Fig. 8). These results suggest that RpMR1 positively regulates components of the TLR pathway during the immune response (Fig. 8).

## Discussion

The *RpMR* genes contain conserved aromatic amino acids, including phenylalanine (Phe) and tryptophan (Trp). Studies have shown that these two amino acids are critical for the *MR* gene to bind to collagen types I, II, III, and IV in the extracellular matrix (Zheng et al. 2015). The *MR* gene can degrade and metabolize endogenous glycoproteins to maintain body stability, while enzymes released from inflammatory sites are recognized by the CLECT domain, which can further decompose and clear them to alleviate inflammatory responses (Zheng et al. 2015). Structural domain analysis revealed that all *RpMR* genes contain CLECT domains, indicating that the immune response of clams induced by *MR* genes may be inseparable from the function of CLECT domains. In this study, we identified 13 mannose receptor genes (*RpMR*1–13) in *R. philippinarum* through genome-wide analysis of publicly available data (BioProject PRJNA479743). Integrated transcriptomic datasets (BioProject PRJNA738278) revealed that six genes (*RpMR*1–6) exhibited differential expression following *V. anguillarum* challenge. The remaining genes (*RpMR*7–13) showed no detectable expression post-infection and were excluded from downstream analyses. We hypothesize that the absence of *RpMR*7–13 transcripts may stem from either: (1) simplified domain architectures reducing functional necessity and mRNA instability (Ibrahim et al., 2020), or (2) excessively complex domain structures hindering transcriptional coordination (Bickmore et al., 2013; Dong et al. 2024), collectively limiting their involvement in antimicrobial immune responses.

According to transcriptomic analysis of clam resistance to *V. anguillarum* stress, we conclude that the *RpMR* gene plays a critical role in the clam's resistance to this pathogen (Dong et al. 2024). In some species, such as *P. trituberculatus* and *C. idella*, the full-length of *MR* gene has been cloned and its immune characteristics analyzed (Wang et al. 2014; Zhang et al. 2019). Functional studies in *P. clarkii* have demonstrated that the *mannose receptor* (*MR*) gene critically determines antimicrobial capacity. *MR* knockdown experiments revealed severe impairment of antibacterial defenses (*P* < 0.05), directly linking its activity to pathogen resistance (Man et al. 2018). Downregulation of *MR* disrupts immune signaling pathways, suppressing inflammatory cytokine production and weakening host antimicrobial responses. Furthermore, *MR* deficiency impairs antigen presentation, attenuating T cell activation and adaptive immunity against *Leishmania* infection. These findings highlight the *MR* play important roles in innate and adaptive immunity (Gazi and Martinez-Pomares 2009). The immunological importance of *MR* genes has been reported in vertebrates and crustaceans, such as *C. idella*, *P. clarkii*, and *E. coioides* (Cano et al.,^ 2024^; Man et al. 2018; Wang et al. 2014; Zhang et al. 2023, 2021). Our study characterized the immune function of the *RpMR* gene in *R. philippinarum* following *V. anguillarum* challenge. Tissue-specific expression analysis revealed predominant *RpMR* expression in the hepatopancreas, with detectable levels in other tissues. This expression pattern parallels the conserved immunological roles of mannose receptors documented in *D. rerio*, *L. hoevenii*, and *P. clarkii* (Gazi and Martinez-Pomares 2009; Man et al. 2018; Zheng et al. 2015).

In this study, mortality analysis revealed that intramuscular administration of recombinant RpMR1 protein significantly reduced *R. philippinarum* mortality by 26% following *V. anguillarum* challenge (*P* < 0.05), demonstrating its potential to enhance anti-*Vibrio* immunity in clams. Complementary studies in *M. amblycephala* have established that the mannose receptor (*MR*) interacts with chitooligosaccharides (COS) to modulate nitric oxide synthase (*NOS*) expression (Ouyang et al. 2021). Importantly, our findings align with recent evidence confirming NOS as a critical component of phagocytic machinery, where it facilitates pathogen clearance through reactive nitrogen species production (Yin et al. 2022b). Emerging evidence establishes nitric oxide (NO) as a pivotal mediator of neutrophil immunity, where inducible nitric oxide synthase (iNOS)-derived NO promotes phagolysosome maturation and enhances microbicidal activity (Kumar et al., 2024). Intriguingly, our experimental data revealed that the NOS activity in the group injected with recombinant RpMR1 protein was significantly increased at 12 h post-*V. anguillarum* stress. This investigation provides the first functional evidence of MR-dependent regulation of NO synthase pathways in bivalve immunity, bridging receptor-mediated recognition with downstream effector mechanisms in molluscan host defense. Our findings elucidate the critical role of the MR in enhancing phagocytic engulfment of *V. anguillarum* by hemocytes. However, the precise molecular interplay by which RpMR1 confers anti-*Vibrio* immunity through NOS-dependent mechanisms remains to be delineated, necessitating targeted investigations to unravel this tripartite interaction between MR activation, effector enzyme regulation, and pathogen clearance.

The antimicrobial efficacy of mannose receptor (*MR*) recombinant protein against Gram-negative bacteria has been well documented (Gazi and Martinez-Pomares 2009; Zheng et al. 2015). While our cell-free in vitro assays exclude cellular components, RpMR1 demonstrates bacterial-binding capacity through PAMP recognition, forming immune complexes that suggest dual mechanisms: 1) priming phagocyte activation via opsonin-like effects, and 2) potentiating complement-dependent bacteriolysis. These findings provide mechanistic insights for future investigations into receptor-mediated immune potentiation in vivo (Huo et al. 2024a). Our study corroborates the antimicrobial specificity of recombinant RpMR1 protein, demonstrating significant growth inhibition against Gram-negative *Vibrio* pathogens, including *V. anguillarum*, *V. splendidus*, and *V. alginolyticus*. This bactericidal activity likely stems from RpMR1's affinity for lipopolysaccharides (LPS), the dominant outer membrane component of Gram-negative bacteria. Notably, LPS serves as a conserved PAMP that is specifically recognized by *TLR4 *to initiate MyD88-dependent signaling cascades—a critical immune activation pathway conserved from invertebrates to mammals (Nunes-Alves 2014). It has been demonstrated that the main mechanism of MR recognition and binding to pathogens is due to the presence of phagocytic cells on the surface, which activates innate immunity through phagocytosis and simultaneously activates pro-inflammatory signaling pathways (Zheng et al. 2015). However, emerging evidence suggests that *MR* lacks intrinsic signaling capacity, requiring cooperative interactions with complement receptor 3 (*CR3*) and Fc receptors to transduce immune activation signals. This receptor clustering mechanism ensures precise control over inflammatory responses while maintaining immune homeostasis (Balderramas et al., 2014; Xaplanteri et al. 2009; Zheng et al. 2015). Cytokine profiling studies reveal a synergistic interaction between *MR* and *TLR4* in orchestrating pro-inflammatory mediator release, particularly *IL-1β*, *TNF-α*, and *IL-6 *(Zhang et al. 2005; Zheng et al. 2015). Mechanistically, MR expression in dendritic cells modulates Th2 cell polarization through C-type lectin receptor (CLR)-mediated antigen presentation. Crucially, TLR4 signaling serves as a critical modulator of this immunoregulatory axis, with stromal cell-specific TLR4 activation being indispensable for initiating allergen-induced Th2 immunity against inhaled particulates (Li et al. 2010; Tan et al. 2010). Our results show that the expression of *TLR4* gene decreased significantly after injection of RpMR dsRNA, demonstrating the potential interaction between *MR* and *TLR* in innate immune response. In addition, we determined the gene expression of TLR signaling pathway after injection of *V. anguillarum* and RpMR1 recombinant protein, with most of the genes in the TLR pathway reaching peak expression at 6 h in the early stage of infection. Mechanistic insights suggest that RpMR1 recombinant protein activates TLR signaling pathways through PRRs, triggering rapid antimicrobial responses against *V. anguillarum* infection. The immunological functions of RpMR1 recombinant protein are anticipated to be applied in the aquaculture of *R. philippinarum*, where it can be incorporated into feed to serve as an antimicrobial agent. However, for large-scale application, further research is needed to determine the optimal dosage of the recombinant protein and its capacity to maintain sustained antibacterial activity.

## Conclusions

In this study, we investigated the function and mechanism of action of mannose receptors in *R. philippinarum* against *V. anguillarum* for the first time. The results showed that recombinant RpMR1 protein significantly reduced clam mortality following *V. anguillarum* infection. Recombinant RpMR1 protein exhibited inhibitory effects on Gram-negative bacteria, including *V. splendidus*, *V. alginolyticus*, and *V. anguillarum*. Furthermore, the protein rapidly activated the TLR signaling pathway, enhancing immune responses through increased nitric oxide synthase (NOS) activity. qPCR analysis revealed that dsRNA-RpMR1 injection suppressed the expression of both *RpMR1* and *TLR4* genes after *V. anguillarum* challenge, indicating a critical regulatory relationship between these immune components. These findings highlight the pivotal role of the mannose receptor in *R. philippinarum*'s defense against *V. anguillarum*, underscoring its importance in bivalve immune defense mechanisms.

## Supplementary Information

Supplementary Material 1: Fig. S1. The intron and exon information of *RpMR* gene family.

Supplementary Material 2: Fig. S2. The distribution of conserved motifs of RpMR protein.

Supplementary Material 3: Fig. S3. The evolutionary relationship and protein domain analysis of RpMR protein.

Supplementary Material 4: Fig. S4. Multiple alignment of RpMR1-13. Red rectangle is labeled as conserved cysteine residue, green rectangle as aromatic amino acid Phe and Trp, blue rectangle as Ca^2+^ binding site, yellow rectangle as conservative acid modified amino acid residue.

Supplementary Material 5: Fig. S5. The chromosomal localization of *RpMR* gene.

Supplementary Material 6: Fig. S6. Phylogenetic tree of MR protein sequences from 9 species.

Supplementary Material 7: Fig. S7. Heat map of *RpMR* gene expression at different developmental periods. Note: Fertilized egg (FE), 1 st polar body (PB1), 2 st polar body (PB2), 2-cell (TC), 8-cell (EC), blastula (B), Gastrula (G), Trochophora (T), D larva (D), Umbo veliger (U), Pediveliger (P), Single pipe juvenile (S), and Juvenile (J).

Supplementary Material 8: Fig. S8. The restriction enzyme map of recombinant plasmid (Digested with NdeI-XhoI).

Supplementary Material 9: Fig.S9. The Western blot method for detecting target proteins.

Supplementary Material 10.

Supplementary Material 11.

## Data Availability

The datasets generated in the current study are available from the corresponding author on reasonable request.
