# Supplementary figures and images for: Mannose receptor RpMR1 of Manila clam (Ruditapes philippinarum) defense against Vibrio anguillarum infection
Source: Adv Biotechnol (Singap). 2025 Aug 4;3(3):23. doi: 10.1007/s44307-025-00075-7 (PMC12321717; doi:10.1007/s44307-025-00075-7)

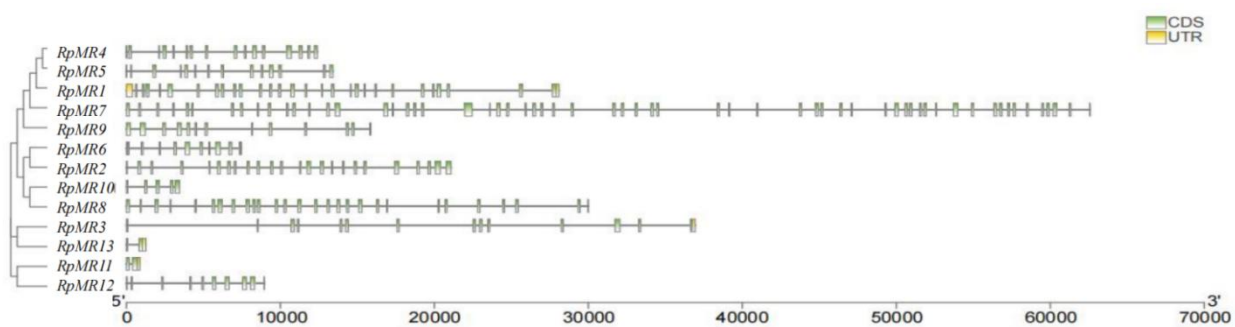

Supplement: Supplementary file 1 — Supplementary Material 1: Fig. S1. The intron and exon information of RpMR gene family. [file 44307_2025_75_MOESM1_ESM.pdf]

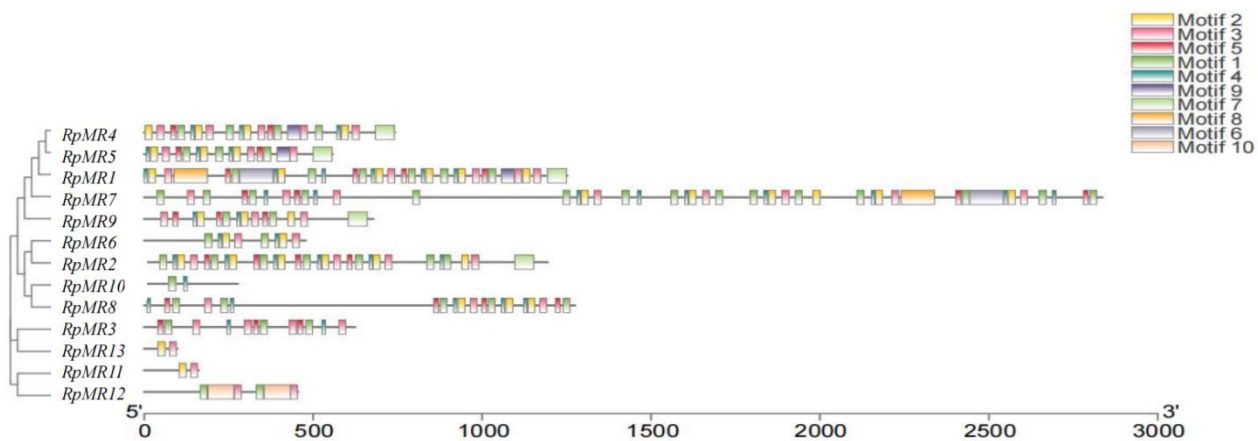

Supplement: Supplementary file 2 — Supplementary Material 2: Fig. S2. The distribution of conserved motifs of RpMR protein. [file 44307_2025_75_MOESM2_ESM.pdf]

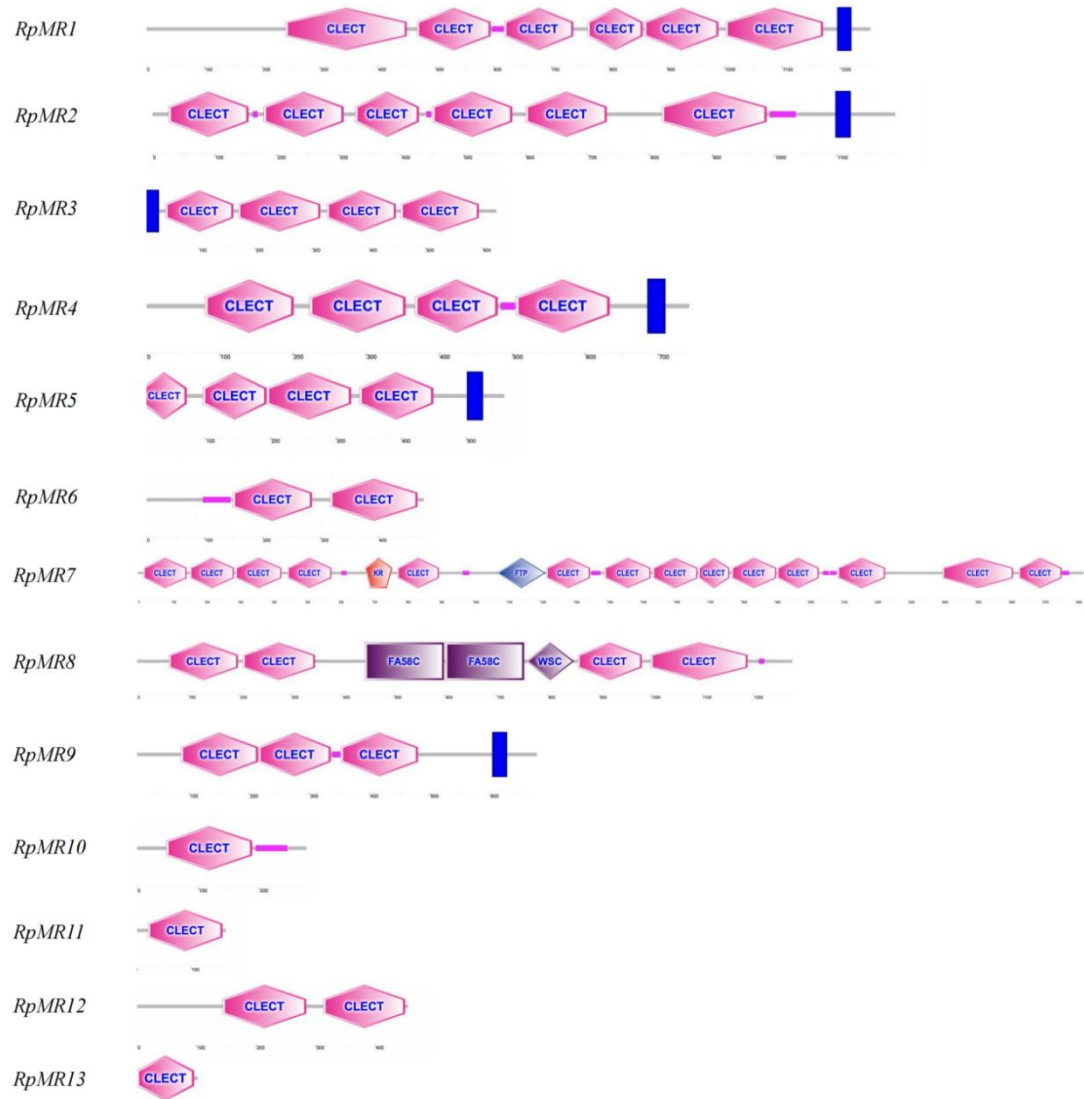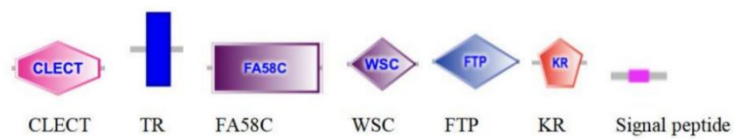

Supplement: Supplementary file 3 — Supplementary Material 3: Fig. S3. The evolutionary relationship and protein domain analysis of RpMR protein. [file 44307_2025_75_MOESM3_ESM.pdf]

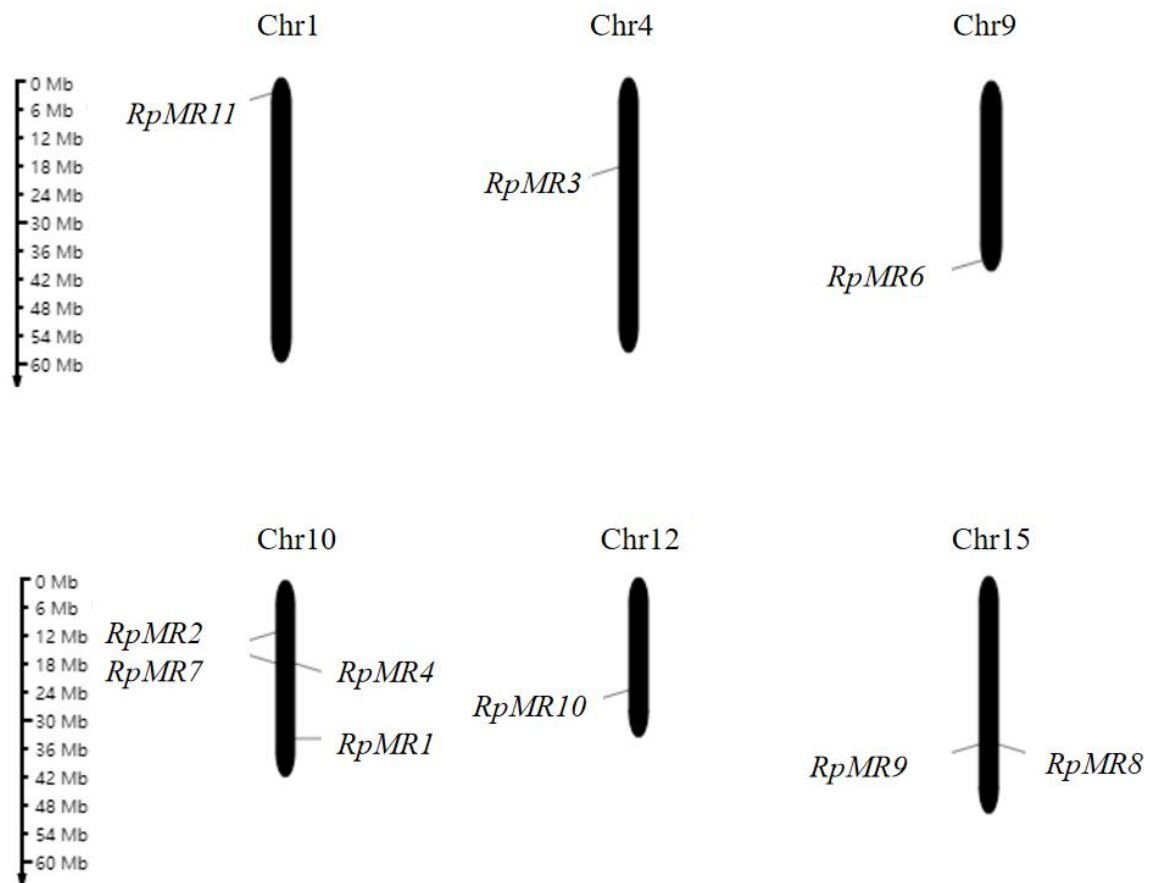

Supplement: Supplementary file 5 — Supplementary Material 5: Fig. S5. The chromosomal localization of RpMR gene. [file 44307_2025_75_MOESM5_ESM.pdf]

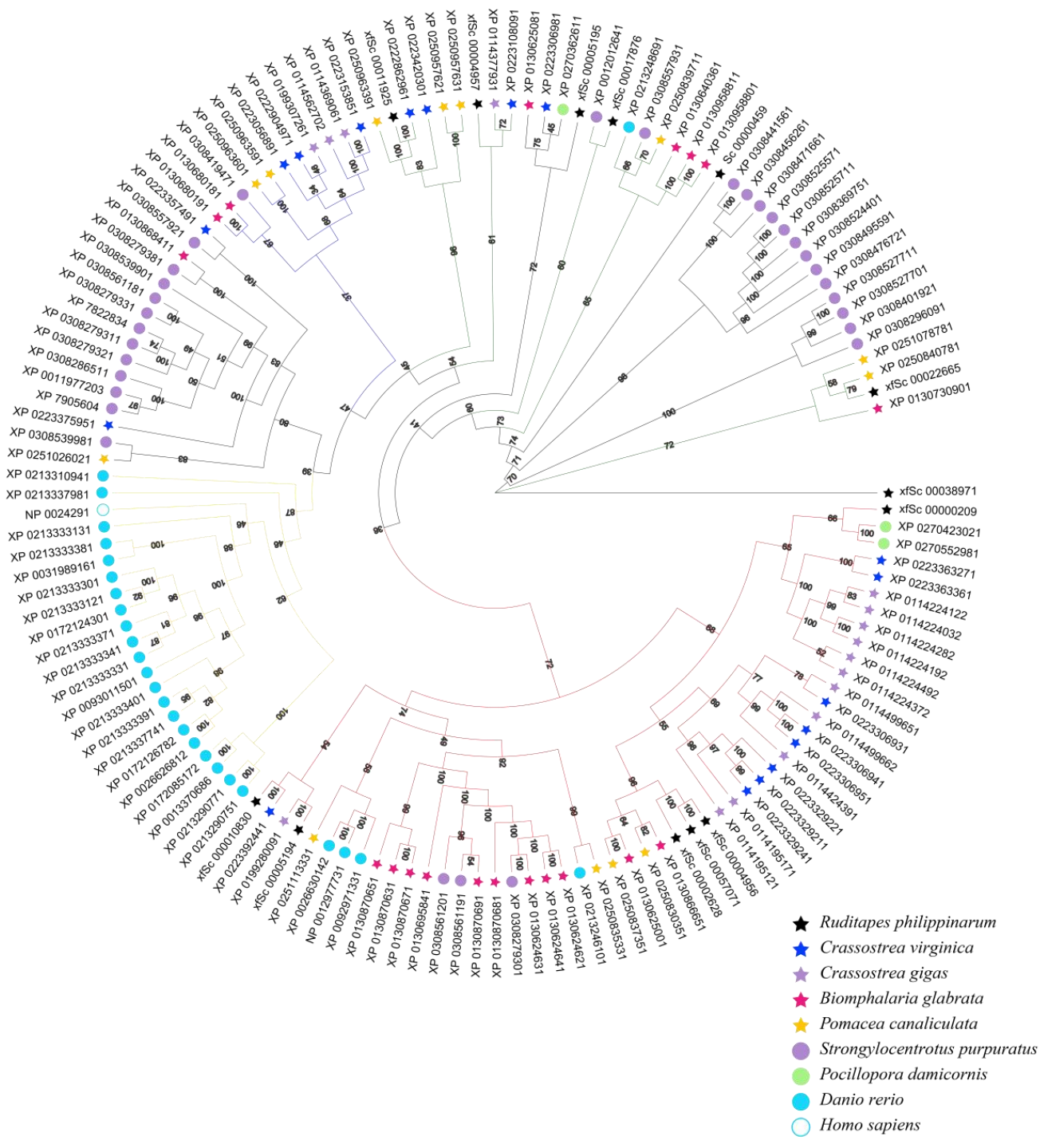

Supplement: Supplementary file 6 — Supplementary Material 6: Fig. S6. Phylogenetic tree of MR protein sequences from 9 species. [file 44307_2025_75_MOESM6_ESM.pdf]

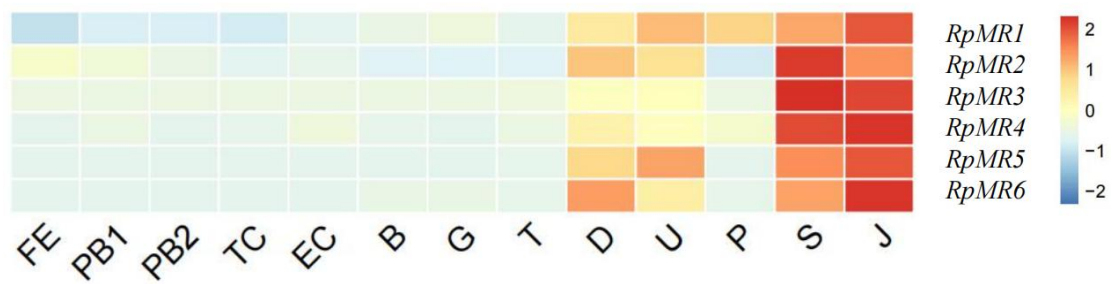

Supplement: Supplementary file 7 — Supplementary Material 7: Fig. S7. Heat map of RpMR gene expression at different developmental periods. Note: Fertilized egg (FE), 1 st polar body (PB1), 2 st polar body (PB2), 2-cell (TC), 8-cell (EC), blastula (B), Gastrula (G), Trochophora (T), D larva (D), Umbo veliger (U), Pediveliger (P), Single pipe juvenile (S), and Juvenile (J). [file 44307_2025_75_MOESM7_ESM.pdf]

bp

12000  
8000  
6000  
5000  
4000  
3000  
2500  
2000  
1500  
1000  
750  
500  
250

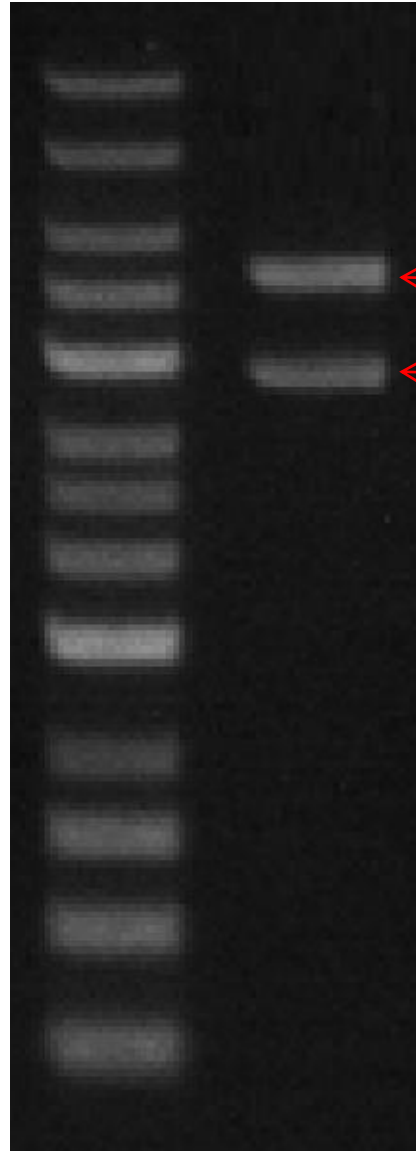

← pET-28a(+)

← RpMR1

1Kb DNA Marker

Supplement: Supplementary file 8 — Supplementary Material 8: Fig. S8. The restriction enzyme map of recombinant plasmid (Digested with NdeI-XhoI). [file 44307_2025_75_MOESM8_ESM.pdf]

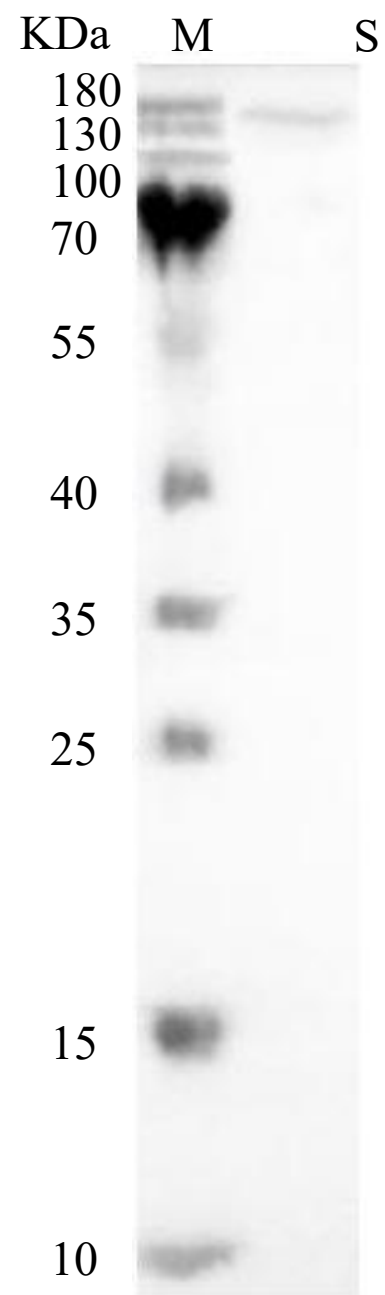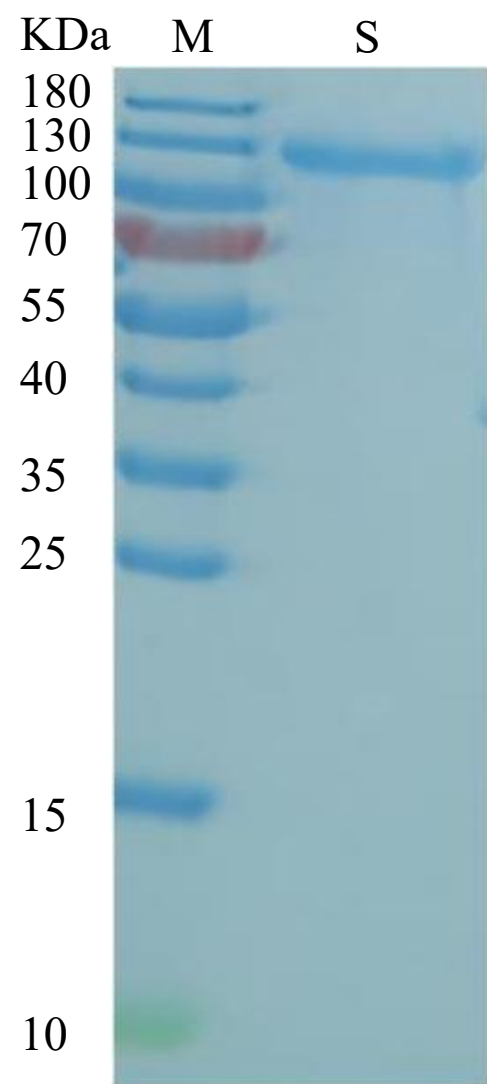

Supplement: Supplementary file 9 — Supplementary Material 9: Fig.S9. The Western blot method for detecting target proteins. [file 44307_2025_75_MOESM9_ESM.pdf]
