# Supplementary material for: Mannose receptor RpMR1 of Manila clam (Ruditapes philippinarum) defense against Vibrio anguillarum infection
Source: Adv Biotechnol (Singap). 2025 Aug 4;3(3):23. doi: 10.1007/s44307-025-00075-7 (PMC12321717; doi:10.1007/s44307-025-00075-7)

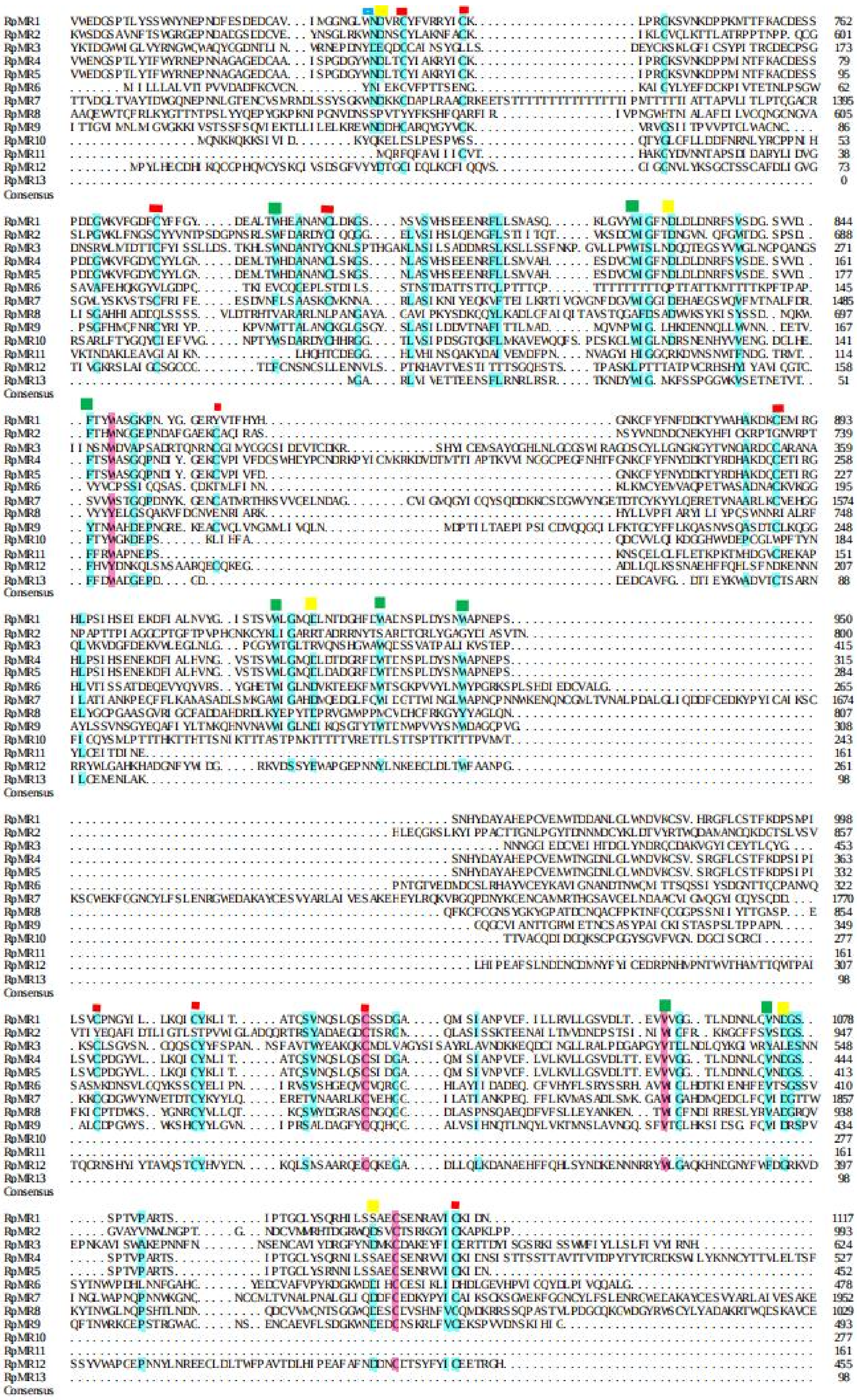

Supplement: Supplementary file 4 — Supplementary Material 4: Fig. S4. Multiple alignment of RpMR1-13. Red rectangle is labeled as conserved cysteine residue, green rectangle as aromatic amino acid Phe and Trp, blue rectangle as Ca2+ binding site, yellow rectangle as conservative acid modified amino acid residue. [file 44307_2025_75_MOESM4_ESM.pdf]
