# Supplementary material for: Mannose receptor RpMR1 of Manila clam (Ruditapes philippinarum) defense against Vibrio anguillarum infection
Source: Adv Biotechnol (Singap). 2025 Aug 4;3(3):23. doi: 10.1007/s44307-025-00075-7 (PMC12321717; doi:10.1007/s44307-025-00075-7)
Supplement: Supplementary file 10 — Supplementary Material 10. [file 44307_2025_75_MOESM10_ESM.docx]

Table S1 Genome of different species

| Species | Number |
| --- | --- |
| *Danio rerio* | GCA_000002035.4 |
| *Homo sapiens* | GCA_000001405.28 |
| *Strongylocentrotus purpuratus* | GCA_000002235.4 |
| *Biomphalaria glabrata* | GCA_000457365.1 |
| *Pomacea canaliculata* | GCA_003073045.1 |
| *Pocillopora damicornis* | GCA_003704095.1 |
| *[Crassostrea gigas](#!/eukaryotes/10758/_blank)* | GCA_902806645.1 |
| *Crassostrea virginica* | GCA_002022765.4 |
| *Ruditapes philippinarum* | GCA_009026015.1 |
