# Supplementary material for: Mannose receptor RpMR1 of Manila clam (Ruditapes philippinarum) defense against Vibrio anguillarum infection
Source: Adv Biotechnol (Singap). 2025 Aug 4;3(3):23. doi: 10.1007/s44307-025-00075-7 (PMC12321717; doi:10.1007/s44307-025-00075-7)
Supplement: Supplementary file 11 — Supplementary Material 11. [file 44307_2025_75_MOESM11_ESM.docx]

Table S2 The specific primers of those genes were listed

| Primer name | Gene ID | Sequence(5′-3′) |  |
| --- | --- | --- | --- |
| *RpMR1* | xfSc0000262.8 | F | GCCAGGAATGTGGACAGATGGTAGC |
|  |  | R | GGACCCTACTCGCCACTGGAATGT |
| *RpMR2* | xfSc0000020.9 | F | GCGGGTCAAGTGGTTGGTATCG |
|  |  | R | TCGCCACCTTGTCGCCTCTT |
| *RpMR3* | xfSc0001192.5 | F | ATACCGGACTGACCGATGAGGG |
|  |  | R | TGGAATCAACCCGCTAGGTGGA |
| *RpMR4* | xfSc0000495.6 | F | ATGGACCGAGTTGCAGACGACT |
|  |  | R | ACACGACGCTGCCAATGATCAA |
| *RpMR5* | xfSc0005707.1 | F | GGACCGAGTTGCAGACGACTCT |
|  |  | R | ACACGACGCTGCCAATGATCAA |
| *RpMR6* | Sc0000045.9 | F | GATTGGGAACGCCAACGACACT |
|  |  | R | TGGACGTTTGCTGGGCATTGAG |
| *β-actin* | AY889707.1 | F | CTCCCTTGAGAAGAGCTACGA |
|  |  | R | GATACCAGCAGATTCCATACCC |
| *MyD88* | xfSc0001099.14 | F | TGAGGACGAGAAAGACCGCCAA |
|  |  | R | GGCAGGTCGTCTCTTCCAGGTA |
| *TRAF* | xfSc0001411.15 | F | TTCGGCGTCGTGCGGAGTAA |
|  |  | R | AGCGTCTGTTCTGCACTTTGGC |
| *NF-κB* | Sc0000074.13 | F | ACGAGCAAGAGATCGTGACACG |
|  |  | R | CCGAGTCGGAATTGTCGTCTCC |
| *IKK* | xfSc0000319.15 | F | GCTCCTGAGCTGTTTGCCAGTC |
|  |  | R | GCGGGCTGTTAGGAAGGAATGG |
| *AP-1* | xfSc0000225.25 | F | CCTCCTGATGCAGACTCTCCCA |
|  |  | R | ACTCCTTGCCTCCAGGGAAAGA |
| *TLR4* | xfSc0000337.32 | F | TCCTAGTTATTCTGCTCCACCA |
|  |  | R | TCACGCATCAAGAGAGTTTCG |
